# Trends in childhood measles vaccination highlight socioeconomic inequalities in Vietnam

**DOI:** 10.1007/s00038-016-0899-4

**Published:** 2016-10-01

**Authors:** Vu Duy Kien, Hoang Van Minh, Kim Bao Giang, Vu Quynh Mai, Ngo Tri Tuan, Mikkel B. Quam

**Affiliations:** 1 Center for Population Health Sciences, Hanoi School of Public Health, No. 138, Giang Vo Street, Dong Da, Hanoi, Vietnam; 2 Unit of Epidemiology and Global Health, Department of Public Health and Clinical Medicine, Umeå University, Umeå, Sweden; 3 Institute for Preventive Medicine and Public Health, Hanoi Medical University, Hanoi, Vietnam

**Keywords:** Socioeconomic inequality, Measles, Vaccine coverage, Children, Vietnam

## Abstract

**Objectives:**

To describe trends in measles vaccine coverage rates and their association with socioeconomic characteristics among children from age 12 to 23 months in Vietnam from the year 2000 to 2014.

**Methods:**

Data were drawn from the Vietnam Multiple Indicator Cluster Surveys in years 2000, 2006, 2011, and 2014. Concentration indices were used to determine the magnitude of socioeconomic inequalities in measles vaccine coverage. Associations between measles vaccine coverage and relevant social factors were assessed using logistic regression.

**Results:**

Socioeconomic inequalities in measles vaccine coverage rates decreased during 2000–2014. Children belonging to ethnic minority groups, having mothers with lower education, and belonging to the poorest group were less likely to receive measles vaccine; although, their vaccine coverage rates did increase with time. Measles vaccine coverage declined among children of mothers with more education and belonging to the wealthiest socioeconomic group.

**Conclusions:**

Understanding the social factors influencing adherence to recommend childhood vaccination protocols is essential. Attempts to regain and retain herd immunity must be guided by an understanding of these social factors if they are to succeed.

## Introduction

Among the most highly contagious infectious diseases, measles can be fatal for children and adults. Measles infection is characterized by fever, cough, conjunctivitis, and eventually rash following 8–12 days of the incubation period (Tannous et al. 2014). Respiratory transmission spreads the virus person-to-person rapidly through populations in close contact with one another, with the peak in infectiousness about 3 days before the onset of rash (Tannous et al. 2014). Due to the similarity of early symptoms with other seasonal viral respiratory infections, rapid spread typically occurs through routine interactions in public places (Perry and Halsey 2004; Tannous et al. 2014). Further complicated control efforts, infected individuals may only seek health care around the peak of infectiousness, often making the hubs of measles outbreaks the hospitals and health care centers, along with schools and child care facilities (Dardis 2012; Perry and Halsey 2004; Yuan 1994).

Measles is a leading vaccine-preventable cause of child mortality; although, mortality has been decreasing over the last half century. The disease burden combined with the vaccine’s utility prompted the ‘proportion of 1-year-old children immunized against measles’ to become a key indicator to monitor the progress of the Millennium Development Goal 4 (MDG-4) aiming to reduce childhood mortality (Nmor et al. 2011). The World Health Organization (WHO) inaugurated the Expanded Programme on Immunization (EPI) in 1974 to increase child immunization rates for many vaccine-preventable diseases including measles (Lievano et al. 2012). Rooted in primary health care and based on the Declaration of Alma-Ata, EPI facilitated measles vaccination so successfully that global coverage increased nearly 70 % in 20 years (Jit et al. 2015; Nguyen and Sendall 2014). To combat child mortality in Vietnam, routine measles vaccination for children under one year of age was mainstreamed Vietnam in 1985, after EPI was first introduced in 1981 (Jit et al. 2015). Even 30 years after the measles vaccine was systematically rolled out in Vietnam, an outbreak having approximately 60,000 cases killed 150 children in Northern Vietnam in 2015 (Ministry of Health of Viet Nam and Health Partnership group 2016; Roberts 2015).

Chiefly dependent on childhood immunization programs’ success, controlling measles and other vaccine-preventable diseases are key functions of public health services for low-, middle-, and high-resource settings (Ohga et al. 1992). Both efficacious and economical, the measles vaccine is also among the safest vaccines used in routine child immunization (Thompson and Odahowski 2014). The vaccine is most commonly administered as a live-attenuated vaccine scheduled for children in the first 2 years of life. Despite the safe, cost-effective and efficacious vaccination options, measles remains a major source of child mortality, especially in low-resource settings with lower coverage rates (An et al. 2016; Jit et al. 2015). Measles remains so communicable that even higher resource settings with higher coverage rates still experience occasional outbreaks, particularly among children (Lee et al. 1998; Plans 2013).

Current recommendations from the WHO and Vietnamese authorities encourage vaccination of all children during the months following birth. Introduced after 2006 in Vietnam, as part of national strategies to eliminate the disease by 2012, was the second dose of measles vaccine around the entry of school, at age 6 (Nguyen and Sendall 2014). In theory, these recommendations would lead to vaccination rates high enough to sustain herd immunity. Coverage rates among youth were said to have been above 90 % since 1992, as high as 95 % in 2006, including the second dose strategy (Nguyen and Sendall 2014). Overall Vietnam’s national vaccination strategies rooted in EPI during the last 30 or so years has been an immense achievement. Some modeled estimations even suggest as much as 96 % of expected measles deaths were avoided due to the successful implementation of childhood immunization (An et al. 2016; Jit et al. 2015).

Like in other settings recently, adherence to recommendations regarding measles vaccination in Vietnam has been waning, resulting in disastrous consequences (Nmor et al. 2011; Pham et al. 2014; Sniadack et al. 2011). Unfortunately, in Vietnam, as more notably studied in the United States, measles vaccine refusal and otherwise under-utilization can be clustered and linked with other social factors influencing inequalities in health (Phadke et al. 2016). Maternal to child transfer of immunity against measles can be an important source of potentially life-saving antibodies; however, these may also be unequally distributed throughout society (Perry and Halsey 2004). If mothers are exposed or vaccinated, their newborn children have brief immunity even before vaccine administration; however, not all children experience such protection, nor equally benefit from maternal immunity.

Some of the factors influencing this inequality are known to fall along social and/or economic gradients. If some of these very young children are also disadvantaged such that many around them are also unvaccinated, or if further complications including malnutrition, vitamin-A deficiencies, and other immune compromising factors accumulate, neither maternal-, self-, nor herd immunity may offer sufficient protection from measles (Fox 1983; Jones 2013). If these factors tend to be socially determined, they may cluster; therefore, increasing the potential for outbreaks in some areas, compared to more homogenously mixed populations, upon which herd immunity theory rests (Black 1982). If some areas, settings, or groups, therefore, have effectively lower coverage rates, transmission can occur in epidemic proportions and quickly overburden health care facilities, and result in many children’s death (Schlenker et al. 1992). To contribute to the prevention of these avoidable sources of child mortality in Vietnam, in this study, we set out to uncover trends and changes in inequalities for measles vaccine coverage among 12–23-month-old children in Vietnam from year 2000 to year 2014.

## Methods

### Sources of data

Data were derived from the Multiple Indicator Cluster Survey (MICS) 2000, 2006, 2011 and 2014 (General Statistics Office (GSO) 2000, 2006, 2011, 2014). The MICS was designed by the United Nations Children’s Fund (UNICEF) to collect internationally comparable data for women and children. In Vietnam, the MICSs were carried out by the General Statistics Office of Vietnam (GSO) with financial and technical support from UNICEF and the United Nations Population Fund (UNFPA). Technical information concerning the survey sampling design and non-response of these MICSs is provided elsewhere (General Statistics Office (GSO) 2000, 2006, 2011, 2014). The numbers of children included in this study in 2000, 2006, 2011 and 2014 were 540, 554, 760 and 785, respectively.

### Variables

In the MICSs, information on vaccination of children was collected either from vaccination cards or by asking mothers. If a vaccination card were available, interviewers would copy the dates on which the child received measles vaccine. If measles vaccination was not indicated on the vaccination card, the interviewers would ask whether the child received a vaccination against measles. The main outcome variable in this study was the binary variable of vaccination status; specifically, whether or not the child, aged 12–23 months at the time of the survey, received measles vaccine when the child was at least 9 months of age but before her/his first birthday. For independent variables, we included child’s age (months), child’s sex, living areas (urban/rural), ethnicity (minorities/Kinh and Hoa), mother’s education level and the household socioeconomic status. Safe water and sanitation were not included in the model because these variables were used to construct the wealth asset.

### Measurement of socioeconomic status

A wealth asset index was used as a proxy for socioeconomic status in this study. The wealth asset index was constructed by principal components analysis (PCA) using information on the ownership of consumer goods, dwelling characteristics, water and sanitation, and other characteristics that are related to the household’s wealth. The estimation method of wealth asset index has been described in detail elsewhere (General Statistics Office (GSO) 2000, 2006, 2011, 2014). The household wealth scores were ranked and also divided into five quintiles equivalent to five socioeconomic groups of equal size (from the poorest group to the richest group).

### Measurement of socioeconomic inequality

We calculated the ‘concentration index’ to measure the degree of socioeconomic inequality in measles vaccine coverage among 12–23-month-old children (O’Donnell 2008; Wagstaff et al. 1991). O’Donnell et al. described the formula for concentration index (O’Donnell 2008) as follows:

1 $$C = \frac{2}{\mu }{\text{cov}}\left( {h,\;r} \right)$$

here,* µ* is the overall percentage of measles vaccination, while h represents the values for measles vaccination of each observation, and r indicates the rank of the household socioeconomic status. The concentration index of measles vaccine coverage among 12–23-month-old children could range between −1 and +1. The concentration index takes the value of 0, if the distribution of measles vaccine coverage among 12–23-month-old children is completely equal between the children of wealthier and poorer families. If it is negative, it indicates that the concentration of measles vaccine coverage among 12–23-month-old children is higher among those children in poor families, and if it is positive, it indicates that the concentration of measles vaccine coverage among 12–23-month-old children is higher among those children in wealthier families (O’Donnell 2008). Household socioeconomic status was also used in the continuous form to increase the precision of estimation.

### Statistical analysis

For each survey period, the percentage of measles vaccination among 12–23-month-old children before the first birthday was estimated overall and according to sex, area (rural and urban), ethnicity (minority and Kinh/Hoa), mother’s education and socioeconomic status. Comparing measles vaccination between 2014 and 2000 was presented as percentage and 95 % confidence interval. In addition, we used the command “igini” of the Distributive Analysis Stata Package (DASP) (Araar and Jean-Yves 2007) to calculate the concentration index of measles vaccination. We also used the command “digini” to generate results of a statistical test on whether the concentration index was significantly statistically different from zero using independent two-tail *t* test, and to later assess the difference between concentration indices of measles vaccination between 2014 and 2000. Multivariable analysis was conducted with logistic regression for the binary outcome variables of whether 12–23-month-old children received measles vaccination or not. All statistical analyses were carried out using Stata^®^ 13.1, with weighting factors for children from the dataset. The level of statistical significance was set to 0.05.

## Results

### Trends in measles vaccine coverage

**Fig. 1:**
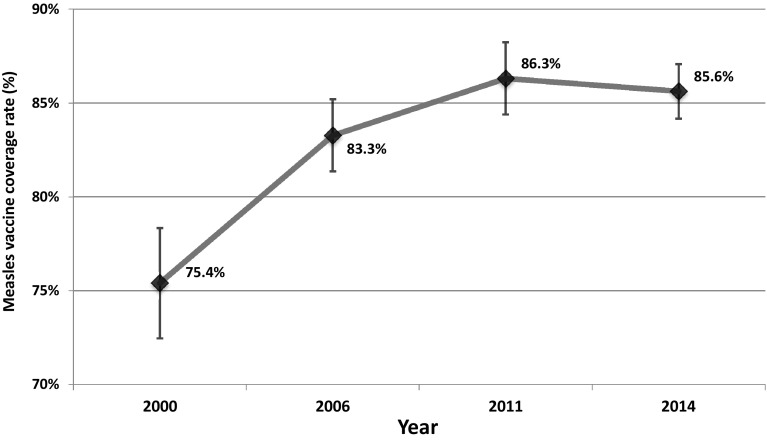
Trends in overall measles vaccination coverage rates among 12–23-month-old children, receiving vaccination before their first birthday in Vietnam, 2000–2014

Figure 1 shows measles vaccine coverage among 12–23-month-old children, who were vaccinated before the first birthday between 2000 and 2014. The percentage of measles vaccination increased significantly between 2000 and 2011, but slightly decreased between 2011 and 2014. In 2014, the measles vaccine coverage was 85.6 %. Trends in measles vaccine coverage in different socioeconomic groups are shown in Table 1. Most of the socioeconomic groups experienced improvements in measles vaccine coverage, especially 12–23-month-old children in rural area, belonging to minority ethnicities, having mothers’ education at primary level or less, and belonging to the poorest group. However, the measles vaccine coverage among 12–23-month-old children decreased significantly in urban areas, among those whose mothers’ education is at upper secondary or tertiary levels, and belong to the richest group.

**Table 1:** Measles vaccination coverage rates among 12–23-month-old children in Vietnam by different social factors, 2000–2014

	Measles vaccination coverage rate % (SE)				Change from 2000 to 2014 % (95 % confidence interval)
	Year 2000 (*N* = 540)	Year 2006 (*N* = 554)	Year 2011 (*N* = 760)	Year 2014 (*N* = 785)	
Sex of children					
Female	75.9 (3.0)	83.9 (2.7)	85.3 (2.3)	85.3 (2.0)	9.4 (2.1–16.7)
Male	75.0 (3.7)	82.8 (2.7)	87.2 (2.4)	85.9 (1.9)	11.0 (2.9–19.0)
Area					
Rural	71.3 (3.2)	81.4 (2.2)	86.1 (2.5)	87.1 (1.7)	15.8 (8.7–22.9)
Urban	91.3 (2.9)	90.1 (3.3)	86.7 (2.8)	82.4 (2.7)	−8.9 (−16.8 to −0.1)
Ethnicity					
Minorities	51.6 (6.9)	69.8 (4.6)	77.6 (5.1)	75.7 (3.8)	24.1 (8.5–39.8)
Kinh/Hoa	81.0 (2.7)	86.3 (2.0)	87.8 (2.1)	87.4 (1.5)	6.4 (2.3–12.5)
Mother’s education					
Primary or less	64.2 (3.9)	78.6 (3.3)	75.3 (4.8)	77.9 (4.0)	13.7 (2.8–24.6)
Lower secondary	80.4 (3.3)	84.0 (2.6)	89.2 (2.5)	88.8 (2.1)	8.3 (0.5–16.2)
Upper secondary and tertiary	90.3 (3.8)	92.6 (3.0)	88.7 (2.4)	86.1 (1.9)	−4.2 (−12.7 to 4.2)
Socioeconomic status (quintile)					
Poorest, 20 %	51.9 (5.0)	69.6 (4.3)	81.5 (4.3)	76.1 (3.7)	24.1 (11.8–36.5)
Near poor, 20 %	80.0 (3.4)	80.4 (4.6)	90.2 (3.0)	90.2 (3.0)	9.4 (5.5–18.3)
Middle, 20 %	88.2 (3.6)	83.9 (3.7)	90.8 (3.4)	92.0 (2.5)	3.8 (−4.7 to 12.4)
Richer, 20 %	78.6 (5.3)	87.9 (3.4)	84.5 (3.5)	89.7 (2.8)	11.1 (−0.1 to 22.4)
Richest, 20 %	98.1 (1.2)	93.4 (2.6)	85.7 (3.5)	80.0 (3.8)	−18.2 (−26.1 to −10.3)
Total	75.4 (2.9)	83.2 (1.9)	86.3 (1.9)	85.6 (1.5)	10.2 (3.8–16.6)

*SE* standard error

### Trends in socioeconomic inequalities in measles vaccine coverage

**Fig. 2:**
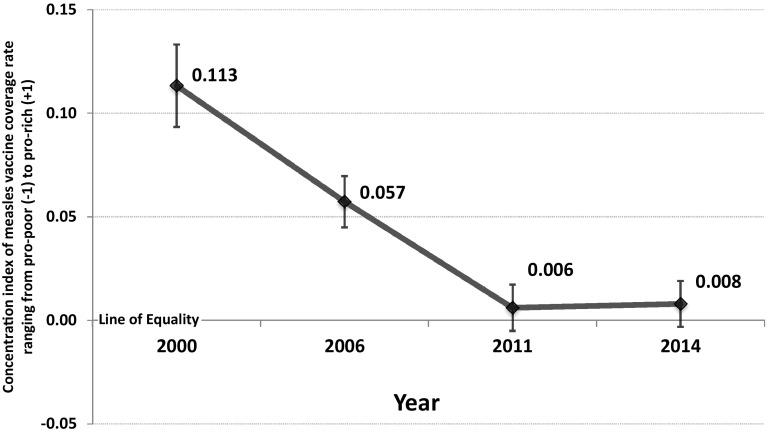
Trends in concentration indices of measles vaccination coverage among 12–23-month-old children in Vietnam, 2000–2014

In Fig. 2, there is a clear decreasing trend in the concentration indices of measles vaccine coverage among 12–23-month-old children during the period between 2000 (concentration index = 0.133) and 2014 (concentration index = 0.008). The concentration indices of measles vaccine coverage reduced significantly from 0.113 in 2000 to 0.006 in 2011, but increased slightly from 0.006 in 2011 to 0.008 in 2014. All values of the concentration indices were positive, indicating that the children in wealthier families had a higher likelihood to receive measles vaccination. Table 2 shows trends in the concentration indices of measles vaccine coverage among 12–23-month-old children in different socioeconomic groups. In 2000 and 2006, most of the concentration indices were positive and significantly different from 0, indicating that the measles vaccine coverage was more likely to favor the rich children over the poor children. The concentration indices in 2011 were not significantly different from 0, indicating that there was no clear inequality in measles vaccine coverage along the wealth gradient. However, in 2014, the concentration indices were positive and significantly different from 0 among 12–23-month-old children in rural areas, belonging to ethnic minority groups, and born to mothers with primary or lower education. Therefore, the measles vaccine coverage was more likely to favor the children in wealthier families within these groups.

**Table 2:** Concentration indices of measles vaccination among 12–23-month-old children in Vietnam by different social factors, 2000–2014

	Measles vaccination coverage rate Concentration index (SE)				Change from 2000 to 2014 (95 % confidence interval)
	Year 2000 (*N* = 540)	Year 2006 (*N* = 554)	Year 2011 (*N* = 760)	Year 2014 (*N* = 785)	
Sex of children					
Female	0.134***	0.054**	0.010	0.003	0.130 (0.070–0.191)
Male	0.094***	0.061***	0.003	0.012	0.082 (0.023–0.141)
Area					
Rural	0.110***	0.068***	0.009	0.033*	0.077 (0.026–0.127)
Urban	0.045*	0.001	0.000	−0.015	0.060 (0.001–0.120)
Ethnicity					
Minorities	0.058	0.058	0.032	0.093***	−0.034 (−0.147 to 0.078)
Kinh/Hoa	0.076***	0.036**	−0.011	−0.018	0.093 (0.053–0.133)
Mother’s education					
Primary or less	0.092*	0.068**	0.057	0.112***	−0.020 (−0.112 to 0.071)
Lower secondary	0.072**	0.043*	−0.016	0.021	0.051 (−0.003 to 0.105)
Upper secondary and tertiary	0.058	0.139	−0.019	−0.041*	0.098 (0.031–0.166)
Total	0.113***	0.057***	0.006	0.008	0.105 (0.061–0.150)

*SE* standard error

*, **, *** Significant at 0.05, 0.01 and 0.001, respectively (*t* test to compare the concentration index with 0)

### Social factors associated with measles vaccination

Table 3 shows the social factors associated with measles vaccine coverage among 12–23-month-old children. After adjusting for other factors, the increase in measles vaccine coverage was significant in 2006, 2011 and 2014 as compared to 2000 (all *p* value <0.05). The main factors that showed significant association with lower measles vaccine coverage among 12–23-month-old children were children belonging to ethnic minority groups, having mothers with education at the primary level or less, and belonging to the poorest groups, even though some progress had been made over the study period among certain disadvantaged groups.

**Table 3:** Social factors of measles vaccination coverage among 12–23-month-old children in Vietnam, 2000–2014: multivariable logistic regression analysis

	Measles vaccination coverage rate	
	OR (95 % confident interval)	*p* value
Year		
2000	1	
2006	1.6 (1.1–2.3)	0.03
2011	1.8 (1.1–2.9)	0.02
2014	1.6 (1.1–2.4)	0.01
Child’s age (months)	1.0 (0.99–1.1)	0.10
Sex of children		
Female	0.9 (0.8–1.2)	0.61
Male	1	
Area		
Rural	1.1 (0.8–1.5)	0.65
Urban	1	
Ethnicity		
Minorities	0.7 (0.5–0.9)	0.02
Kinh/Hoa	1	
Mother’s education		
Primary or less	0.6 (0.4–0.9)	<0.01
Lower secondary	0.9 (0.7–1.3)	0.74
Upper secondary and tertiary	1	
Socioeconomic status (quintile)		
Poorest, 20 %	0.5 (0.3–0.9)	0.04
Near poor, 20 %	1.1 (0.7–1.8)	0.75
Middle, 20 %	1.3 (0.8–1.5)	0.28
Richer, 20 %	0.9 (0.6–1.5)	0.78
Richest, 20 %	1	

*OR* odds ratio

## Discussion

We found that poorer, lower educated mothers belonging to ethnic groups were less likely to have taken the opportunity to have their children vaccinated over the time period 2000–2014 compared to their socially and economically advantaged counterparts in Vietnam. This finding, importantly, is likely to be clustered along with the residential settlement patterns and health seeking behavior of like-groups, such that the under-vaccinated profile may be congregated together and represent potential gaps in herd immunity in some geographical pockets and among some social factions. The tendency for disadvantaged groups to have unvaccinated children represent a multiple disease burden for both individuals and their families, which can spark an epidemic of measles and related economic depression and even the death of children within already marginalized settings, both rural and urban.

Although not all trends for all groups were found to be significant, those in the highest wealth category, those with the highest education and those living in urban areas tended to decline in their collective adherence to vaccination recommendations at a higher percentage than most other groups over the 15-year time period of this study. This recapitulates recent trends in high-income settings to an extent, where increasingly large subsets of society refuse to vaccinate their children for non-medical reasons (Phadke et al. 2016). Similarly, a recent systematic review, including studies in 19 low- and middle-income countries, found vaccine hesitancy for individuals and communities to be related to concerns about harmful effects, mistrust, and health system access issues (Muñoz et al. 2015). In some low-income countries, rural populations, ethnic minorities, and poor populations have greater vaccine hesitancy, as was the case with Vietnam toward the beginning of the study. However, other countries, like Vietnam, toward the end of study period have shown wealthier, higher educated, more urbanized populations to be clusters of vaccine hesitancy, similar to high-income countries. Reasons, for which groups and individuals are vaccine hesitant along with solutions to increase vaccine coverage, are context specific and tend to be clustered, consistent with our findings in Vietnam (Dubé et al. 2015; Muñoz et al. 2015).

The combined trends of the relatively disadvantaged subgroups increasing measles vaccination rates with the decreasing rate of the more privileged subgroups throughout the study resulted in a peak in overall vaccine coverage occurring in 2011 at 86.3 %. As rates fell in 2014 to 85.6 and 82.4 % in urban areas, Vietnam’s vulnerability to measles outbreaks increased as higher proportions of wealthy, highly educated mothers tended to decide against measles vaccination. Albeit rarely, outbreaks do occur and children infected with measles virus cluster with those who are unvaccinated and still susceptible, as with the tragic 60,000 case outbreak centered in Hanoi (northern Vietnam) in 2014 (Roberts 2015). During this outbreak alone 150 children died, at least some of whom likely contracted the virus in hospitals, which often become hubs for measles transmission. The occurrence of this tragic measles outbreak provides further evidence of the urgent need for increased vaccine coverage to prevent future epidemics.

Due to the characteristically high communicability, immunization coverage rates and vaccine efficacy needed for herd immunity are much higher for measles than less infectious diseases (Plans 2013). Classically, the calculations for the needed vaccination rate have depended on more static population models, where new births were the main source of introduction of new susceptible persons into the population (Black 1982). Recently, in many settings including Vietnam, measles vaccination has been waning, while simultaneously urban populations have become more dynamic in terms of in/out migration (Gay et al. 1995; Lewis et al. 2015; Phadke et al. 2016). The multiple introductions of virus from imported infections combine with clustering of individuals, who have not been adequately immunized against the virus to create large-scale outbreaks, which can be fatal even within largely high coverage nations (Kremer et al. 2007; Liffick et al. 2001; Phadke et al. 2016). For this reason, the elimination and eventual eradication of measles would require at least 90 % vaccine coverage rates to provide the necessary herd immunity to prevent outbreaks from proceeding (Lee et al. 1998; Plans et al. 2014).

Due to the immense resources and prioritization of routine childhood vaccination strategies employed by Vietnam’s EPI, our study clearly showed increases in the uptake of vaccination among 12–23-month-old children in Vietnam. This, like the success of the previous 15 years since the measles vaccine was rolled out in Vietnam, has no doubt saved many children’s lives. While our study showed increased vaccine coverage, we did not find as high of coverage as EPI, likely in part due to our definition, that the included children received vaccine between 9 and 12 months of age. Still, overall during this period, the increase in coverage of vaccination rates among our study population represents a significant reduction in inequalities between different socioeconomic groups. However, consistent with other studies, mistrust in vaccine safety, lack of confidence in the governmental bodies administering the vaccine, and fears about adverse reactions all contribute to some groups’ decreased participation in child immunization programs (Ali et al. 2005; Dubé et al. 2015; Roberts 2015). At the same time as privileged groups declined in coverage rates, the poorest, least educated, ethnic minorities living in rural areas made substantial gains in vaccine coverage, but remain far below their dominant group, more educated, urban, and middle-income counterparts. As coverage increased, socioeconomic inequalities decreased and as coverage decreased, socioeconomic inequalities increased. If these frightening trends continue to hold, coverage rates among the disadvantaged at relatively low levels, and the privileged continue to diminish the value of vaccination, overall coverage levels may again decline and so too socioeconomic inequalities may resurge further exacerbating the children’s health burden of measles in Vietnam.

More research is needed to identify strategies for targeting the continued elevation of participation in vaccine-preventable disease immunization programs such as EPI in Vietnam to continue to close the gaps of inequality, which accounts for numerous childhood deaths resultant from measles infection. Our findings suggest such strategies may be key in elevating the overall early childhood vaccination against measles, needed to prevent measles deaths in Vietnam. Although working to maintain momentum with the most socioeconomically disadvantaged groups may raise the average coverage the most, a refined strategy targeted on the other extreme of the socioeconomic spectrum will be needed to maintain and sustain long-term herd immunity, as well (Lanphear 1992). The socioeconomic inequalities association with vaccine coverage rates supports more targeted approaches to population subgroups. Efforts, therefore, should be strengthened to continue coverage rate increases among children of mothers with low education, of ethnic minorities, and the least wealth. Simultaneously, further and increased efforts are needed among the more educated, urban wealthiest mothers to reverse the trend of more privileged families’ opting out of measles vaccination in Vietnam. Perhaps targeted social marketing campaigns could be employed to counter misinformation about vaccination safety and scheduling. Furthermore, staff trainings, addressing potential reasons for vaccine refusal, could be conducted to increase mothers’ trust in those administering measles vaccine as suggested by Nguyen and Sendall in their 2014 qualitative study (Nguyen and Sendall 2014).

We acknowledge some limitations to this study. First, due to the limitation of the cross-sectional study design of the MICS, the results should be interpreted with caution so that they are not interpreted as implying causality. Second, the estimations of measles vaccination were derived from available information on vaccination cards and reported by mothers if their children got the measles vaccination. Therefore, there exists the possibility that some children may have been vaccinated, but did not have immunization cards and were excluded from the study. In addition, mothers may have forgotten to report the measles vaccination of their children during their interviews. These would underestimate the measles vaccine coverage in this study. Finally, unknown factors that were not included in our analysis may also be associated with the measles vaccine coverage.

Despite these weaknesses, the findings from this study have some meaningful policy implications. First, this study confirmed that there were some trade-offs between the increase in measles vaccine coverage and the reduction of socioeconomic inequalities. In addition, this study identified vulnerable population groups, upon which the policy makers should focus their efforts to equitably and sustainably tackle the inequalities in the measles vaccine coverage and promote long-lasting herd immunity in Vietnam.

In conclusion, we have shown that, despite much progress in the overall improvement of measles vaccination adherence in Vietnam between 2000 and 2014, measles vaccine coverage rates are largely socially determined; as such, so too are the subsequently related morbidity and mortality of measles. Although, expansions of vaccine coverage among less privileged groups are encouraging and potentially contributing to greater equity in health outcomes, startling recent inclinations among highly educated wealthy urban populations may prolong the measles elimination timeline in Vietnam. These inter-related realities of social position and under-vaccination should be carefully considered in the generation and promotion of health policy seeking to prevent in future the spread of measles within the country and its heterogeneous populations. Tailored strategies, which can address both sides of the socioeconomic spectrum, should be a part of the ongoing campaign for measles immunization in Vietnam.
